# Genomics and Newborn Screening: Perspectives of Public Health Programs

**DOI:** 10.3390/ijns8010011

**Published:** 2022-01-28

**Authors:** Aaron J. Goldenberg, Roselle Ponsaran, Amy Gaviglio, Dalton Simancek, Beth A. Tarini

**Affiliations:** 1 Department of Bioethics, Case Western Reserve University School of Medicine, Cleveland, OH 44106, USA; rsp10@case.edu; 2 G2S Corporation/CDC Newborn Screening and Molecular Biology Branch, Atlanta, GA 30341, USA; pxd9@cdc.gov; 3 Child Health Evaluation & Research (CHEAR) Unit, Division of General Pediatrics, University of Michigan, Ann Arbor, MI 48109, USA; dsimancek@gmail.com; 4 Children’s Research Institute, Children’s National Health System, Washington, DC 20012, USA; btarini@childrensnational.org

**Keywords:** newborn screening, genomic testing, next generation genomic sequencing

## Abstract

This study assesses the benefits and challenges of using genomics in Newborn Screening Programs (NBS) from the perspectives of State program officials. This project aims to help programs develop policies that will aid in the integration of genomic technology. Discussion groups were conducted with the NBS Program and Laboratory Directors in the seven HRSA Regional Genomics Collaboratives (August 2014–March 2016). The discussion groups addressed expected uses of genomics, potential benefits, and challenges of integrating genomic technology, and educational needs for parents and other NBS stakeholders: Twelve focus groups were conducted, which included participants from over 40 state programs. Benefits of incorporating genomics included improving screening modalities, supporting diagnostic procedures, and screening for a wider spectrum of disorders. Challenges included the costs of genomics, the ability to educate parents and health care providers about results, and the potential negative psychosocial impact of genomic information. Attempts to address the challenges of integrating genomics must focus on preserving the child welfare goals of NBS programs. Health departments will need to explore how genomics could be used to enhance programs while maintaining universal access to screening.

## 1. Introduction

Over its 50-year history, the expansion of newborn screening (NBS) has been fueled by the development of new testing technologies. In accordance with this history, there is now a growing interest in utilizing next generation genomic sequencing (NGGS) for a variety of NBS purposes from sequencing of a single gene, to creating sequencing panels of a state’s entire set of screened conditions, to sequencing a whole exome or a whole genome [1]. There are a number of potential benefits to integrating NGGS into NBS programs. As an adjunct to current screening tests, NGGS may help NBS programs identify specific genotyps, or pathogenic variants, that could shorten a family’s “diagnostic odyssey” after a positive screen [2]. While its benefits are currently limited to a few conditions, a recent paper by a group of NBS experts highlighted that more genomic information may increasingly help providers to refine potential treatment plans by giving them “more accurate genotype-phenotype information” to inform prognoses, such as “severity of disease or age of onset” [2]. The implementation of NGGS would also allow programs to screen newborns for a much wider range of conditions, including risks for later-onset or chronic conditions.

Alternatively, the use of NGGS in NBS will present a number of programmatic and policy challenges for NBS programs [3,4,5]. For example, genomic sequencing platforms in NBS could represent a dramatic shift in the kinds of information generated through this public health program. The uses of NGGS in clinical settings have already highlighted the difficulties involved in managing, interpreting, and communicating to patients the large amounts of data generated through these sequencing platforms [6]. From a programmatic and policy perspective, NBS programs will have to address the long-term management of and access to the vast amounts of genetic data generated from NGGS. NBS programs must balance suppression of certain results, moral obligations to disclose potentially valuable health information to families, and parents’ rights regarding their children’s genomic information [4]. NBS programs also need to consider the potential harms of disclosing findings with uncertain or ambiguous implications for newborns that parents and their health care providers would find exceedingly difficult to interpret [7,8,9]. When a positive NBS result is discovered, parents and PCPs are typically informed of the results and work together to communicate with medical genetics professionals and/or other specialists to coordinate diagnostic steps and future care. In dealing with potentially complex genomic information, NBS programs may need to develop new approaches to these communication processes in order to adequately educate and counsel parents. However, attempts to address these programmatic and policy challenges must be addressed within the existing NBS mission, which is to safeguard child welfare and improve outcomes for newborns and their families.

There have been a number of recent empirical research projects and expert workgroups, that have examined the risks and benefits of using genomic technologies in a newborn population, compared the effectiveness of NGGS to traditional screening modalities, and described the various ethical, social, and practical challenges of genomic NBS [2,10,11,12,13,14,15]. However, none of these projects have explicitly done so from a public health program perspective. This perspective is crucial given that the challenges associated with the integration of NGGS technology into NBS programs may interfere with the successful functioning of the existing system and impair its ability to deliver care to children and their families. We aim to fill this gap by presenting results from a study that assesses key benefits and challenges of integrating NGGS into NBS programs from the perspectives of the State NBS program officials themselves. Identifying these challenges early will enable NBS programs to proactively develop policies and practices to aid the integration of NGGS technology into NBS programs in ways and, in doing so, preserve the effectiveness of the NBS system and, in turn, improve health outcomes for newborns and their families.

## 2. Methods

### 2.1. Participants

Investigators partnered with the seven Regional Genetics Collaboratives (RCs), whose mission is to promote the translation of genetic medicine into public health and healthcare services with the ultimate goal of improving the health of children and their families [16]. Each RC has about 7–8 state NBS program members. These collaboratives represent a diversity of expertise related to genetics and newborn screening from each of the states within that region. These RCs allowed investigators to host focus groups during events such as their regional meetings and conference calls, which are attended by state NBS program officials and other program stakeholders. Leaders of the RCs helped investigators recruit representatives from each of the three stakeholder categories for the focus groups, (1) NBS Program Directors or Coordinators; (2) NBS Laboratory Directors; (3) Newborn Screening Follow-Up Coordinators. This study was reviewed and approved by the Institutional Review Board (IRB).

### 2.2. Discussion Groups

Discussion groups were facilitated by either Dr. Goldenberg or Dr. Tarini and one of the project’s Research Assistants. Research assistants or the non-moderating PI served as notetakers. Consent forms were signed at the beginning of the session if done in person; for focus groups done over the phone, consent was taken verbally. Focus groups were digitally audiotaped and transcribed for analysis. Each focus group lasted approximately one hour. The discussion group guide was developed through a combination of literature reviews on NGGS in NBS and iterative team conversations with our team which includes clinicians, social science and health services researchers, and representatives from state NBS programs. The focus group guide (Table 1) addressed the following major thematic areas: (1) Current or expected uses of NGGS in NBS programs; (2) Potential Benefits and Challenges of Using NGGS in NBS; (3) Educational or communications needs for parents and other NBS stakeholders; and (4) Policy needs for states regarding NGGS in NBS.

### 2.3. Data Analysis

Standard procedures for analyzing qualitative data were employed, based on successive coding passes by two independent coders, beginning with open coding of content at the level closest to the content of the text, and through broader and more analytic codes [16]. All identifying information was removed from the audio and transcripts, and transcripts were imported into Dedoose, a computer program for managing text data [17]. Thematic domains were identified through a process of intense review of transcript data. Every transcript was coded by at least two team members, and a process of iterative group discussions were used to review and settle any discrepancies between coders.

## 3. Results

### 3.1. Participant Characteristics

In total, twelve focus groups were conducted—four in person and the remainder were conducted during over the phone conference calls. The number of participants in each group ranged from 5–25. Over 100 participants were included in all the focus groups, including NBS officials from over 40 state programs. All seven of the RCs were represented by at least one focus group. The following sections detail the primary themes discussed across our discussion groups, and Supplemental Table S1 contains additional representative quoted associated with each thematic area.

### 3.2. Benefits of Using NGGS in NBS

Participants were asked to describe the advantages or benefits of incorporating genomics into NBS. These perceived benefits were mostly related to the possibility of using NGGS technology to improve the current quality or effectiveness of current screening modalities for conditions already screened within state programs.

Reducing Burden of False Positives: For example, many participants discussed the potential for reducing the number of false positives within NBS results. Participants noted that, following up on out of range initial NBS results, many of which turn out to be false positives, can take a great deal of time, and that reducing that delay by using additional technologies would be helpful. One noted their own experience incorporating genomics as a primary technology into testing for a single condition.

*“…we just had so many false-positives and that created so much work both in the lab and for the short-and the long-term follow-up that we had to find ways to you know reduce the false-positive rate, and that kind of helped us do that, but that’s just one condition.”*

Participants also discussed how the use of genomic technologies could also help ease the burden of false-positives for families by reducing the need for additional samples and long waiting times for additional results.

*“And that’s kind of what we started, because I found that having to call families about probably false-positives and having them go get blood drawn, it just creates a lot of burden and then you have all this excess worry. Whereas if we’re able to do the gene study and find no mutations and have a repeat on some of these disorders, then we can feel pretty comfortable that that’s not it.”*

Provision of Detailed Risk Information: Yet another potential benefit a few participants noted was the ability for genomic results to provide more detailed risk information to the family. Some described families that had received sequencing results from private companies. One participant noted: *“…there’s this need from the community also to say ‘We want everything you know about us*”.

Assist with Future Reproductive Decisions: For other participants, genomics in NBS could provide that additional genetic information that may not only help the affected child directly, but also assist the families in making future reproductive decisions.

*“Yeah, and at this point, you know the resources that parents have can be different. So if a child qualifies for Medicaid and some things can be done, they can be done for the child. They reflect back on the parents, but the parents themselves may not have coverage to get their own sequencing done or even you know identification of some kind of abnormal something, and so especially the fathers. It’s like ‘That’s out of the question. We can’t test dad. You will have to pay for it completely.’ At least if you have a more definitive something in the baby, you can you know reflect back at least that amount of knowledge securely to the parents, even if they themselves can’t get the same or afford the same kind of diagnosis-seeking.”*

Screen for Disorders Not Otherwise Possible: While it was not seen as a primary benefit, some participants did discuss the possibility that genomics could allow NBS programs to include a wider spectrum of disorders into the panel via genomics, especially for conditions that would be difficult or impossible to screen with current MS/MS testing modalities.

*“another benefit would be you know maybe the ability to screen for more disorders where right now there’s you know no biochemical assay, for instance, (and) that lends itself to screening on a population basis.”*

Equitable Access to Genomic Services: Finally, a number of participants noted that one benefit of integrating NGGS into state NBS programs would be the potential to support access to genomic services to all newborns in the US. These participants felt that, because one of the foundational goals of NBS was universal access regardless of socioeconomic states, state programs might be in a position to provide more equitable access to genomic services.

*“it’s one of the only places in life where there’s not healthcare [disparities]… that’s our mantra, right, is universal health? The only time in your life you really could get it [genomics], and so where can we fit in there to benefit our population”?*

### 3.3. Challenges of Using NGGS in NBS

Even as they acknowledged the potential benefits, participants were much more inclined to raise concerns about the technical and ethical challenges of incorporating genomic testing into NBS. The challenges and concerns they raised fell along a number of areas.

Impact of Workforce and Budgets: A primary concern for the stakeholders was the impact of genomics on their workforce and on their budgets. While the lab directors described the cost of upgrading equipment and space, and the ability to justify and acquire the larger budgets that would be needed, as a significant barrier, an even greater overall concern was the ability of staff to handle the follow-up and counseling needed to address the information provided by genomic sequencing. One program official noted that “we just don’t have the manpower to provide information to providers and counsel the families.”

### 3.4. Low Genetic Literacy among Public and Providers

In addition to workforce concerns, many participants also felt that their ability to educate both parents and health care providers would pose a significant challenge to increasing the medical utility of genomic information for families. They worried that, given a lack of genetic literacy among the public and health providers, there would be a steep learning curve for being able to effectively understand complex genomic data—especially in the case of whole exome or genome sequencing. One participant stated that “the doctor has to understand the results, and then the patient has to be taught how to understand the results…The general pediatrician may not know what to do or how to describe it, and then once they give the wrong message, it gets perpetuated incorrectly in the patient population”.

Participants anticipated that, for parents and families, the learning curve could be even greater. Additionally, clarity and transparency about the newborn screening process, and what purposes genetic information may be used for, would be even more important to parents making decisions about NBS screening.

*“They are scared of just even the word “DNA” being used, let alone sequencing a whole genome…There are many parents that don’t even know newborn screening happens, or they just remember, ‘Oh yeah, they took some blood, put it on a card.’ The public would have to be very, very educated that this was happening.”*

Lessons of Past Technology Integration: Many participants referenced earlier experiences of technology change–specifically, the introduction of tandem mass spectrometry (MS/MS) into newborn screening. These program officials warned that their experience with integrating MS/MS should serve as a cautionary tale for integrating genomic technology into NBS.

*“I think we should not repeat the mistakes of the past, because I remember when we expanded newborn screening with tandem mass spectrometry, everybody jumped the gun. So, I think we need to be smart in the way that we should gather some information on those conditions, get an idea of when to start treatment, how to follow these patients before we start the [genomic] screening.”*

Participants were asked to consider using genomics as an adjunct rather than replacement technology. Most participants felt that, in most cases, using genomics as a secondary or adjunct test would allow a more gradual introduction which would lend itself to a controlled and specific use of the technology.

*“I think using it as an adjunct technology would kind of ease us into it, so to speak, and you know and help us to gradually adopt it in small doses and build our knowledge and understanding and the capacity to deal with the information…As replacement technology, I feel it’s like really diving into the deep end and just feel very unprepared for that.”*

NGGS as Replacement Technology: Participants were asked to specifically consider the prospect of potentially moving towards whole genome or whole exome sequencing as a replacement technology. Generally, this was especially worrisome for participants who were concerned about number and types of results that would need to be returned to parents—especially in the context of a mandatory screening test.

*“you really can’t put that burden on parents that we have a mandatory test with a bunch of things that we can’t figure out what’s happening with their kids ‘…I mean it’s hard enough making sure that kids are in nurturing families to suddenly throw on them that ‘We’ve mandatorily tested your child for x, y, z. We have no idea what this mean, but good luck with that,’ right…I don’t want to tell a family that ‘Your child has this late onset disorder. There’s nothing we can do for it, and shouldn’t show any symptoms ‘til maybe later on, some muscle weakness or whatever. Have fun. Good luck with your newborn…I’m not going to do that to a parent.”*

Furthermore, participants also worried about how programs would need to decide what kinds of results to return to parents. For programs, this was complicated by unclear guidelines about how to determine which results may be actionable and whether it was appropriate for public health institutions to be making the decisions about which results to return.

*“We need to be very clear about like the definition of an actionable result…we would need some guidelines about ‘What are actionable results…So to understand that just because we can do the test, doesn’t mean we’re prepared to deal with the results, and maybe we shouldn’t, as public health systems.”*

Finally, participants expressed concerns that too broad use of genomics would in fact hurt the original intention of state newborn screening.

*“Clearly, we need to keep screening for things that have safe, effective treatment, that that’s what newborn screening is based on, and that’s finding out about all these other things that are untreatable or unknowable at this point. So, I think that that clearly goes against the ethics of newborn screening.”*

## 4. Discussions and Conclusions

This study found that key NBS program stakeholders perceived a number of structural, technical, and ethical challenges to the integration of NGGS technology into state NBS programs. While participants understood that there may be some compelling benefits to incorporating genomics in their programs, they had significant concerns, particularly around costs and data management, interpretation and communication of results, psychosocial harms associated with uncertain or ambiguous genomic data, and deviation from the core goals of NBS programs. Most notably, participants raised concerns that failure to address programmatic and policy challenges from the integration of NGGS technology into NBS programs could disrupt the functioning and reduce the benefits of these programs for newborns and their families. For example, management of the genomic data could overwhelm the current capacity of NBS programs and lead to an interruption in key functions. Program officials further raised a number of issues related to the impact this information may have on families. First, the public could raise significant concerns about the potential for misuse or unintended harm from genetic data, such as genetic discrimination or possession of genetic information by a government agency. Second, there may be added concerns about giving families information that either would inform parents about their own genetic risk or reveal carrier status or adult onset information about newborns, which may further move beyond the current ethical justifications for population based screening and violate a newborns right not to know certain genetic results that will not be relevant until they reach adulthood. Finally, and most possibly most concerning, the complexity of information or increased uncertainty about results could also prompt parents to opt out of NBS screening altogether, thus negating the potential benefits of screening for their newborns. Addressing these and other as yet unidentified issues requires systematic research about the kinds of genomic information that should be returned to parents and how best to communicate it, as well as how to address public policy concerns about the use of genomic data by a public health program. The international NBS community will need to clarify the important distinctions between adding genomic sequencing into current NBS approaches and a larger paradigm shift to “genomic screening” of newborns which could include whole genome or exome sequencing of all newborns. This shift may further move programs away from their core goals and disrupt the benefits of NBS, especially if the addition of genomics adds significant increases in uncertain results being returned to families. Furthermore, many international NBS stakeholders, including the EUNENBS network of newborn screening experts, have noted the important differences between screening and diagnostic approaches [18], and thus a shift to more diagnostic genomic sequencing may again further move programs away from their primary goals of population based screening.

Another related overarching message from NBS program stakeholders focused on the history of challenges associated with using new testing modalities in NBS programs. Advances in testing technology have always been both the greatest benefit and the greatest challenge to NBS programs’ ability to improve health outcomes for newborns and their families [19]. For example, tandem mass spectrometry (MS/MS) technology allowed programs to screen for more disorders without significantly increasing screening costs. However, the increased number of tests was accompanied by an increase in the number of false positive results [20], inadequate education of primary care physicians about newly-screened disorders [21], and indeterminate results [8]. Many of our participant stakeholders felt that the integration of NGGS technology into NBS programs would pose similar challenges. They noted the need for programs to work together to avoid some of the mistakes of the past, while finding effective ways to utilize new technologies and improve programs [22,23,24].

It seems that, moving forward, it is critical that NBS programs share strategies and lessons as they work to incorporate NGGS into NBS. Such a widespread collaborative approach to complex problems in NBS is not new. In fact, the Collaborative Improvement and Innovation Network (COINN) for Timeliness in Newborn Screening is an example of such an approach to addressing the challenges of ensuring timely collection and processing of NBS specimens. This initiative provides a potentially valuable and useful model for NBS programs as they contend with the challenges of integrating NGGS.

As with all studies, there are limitations that should be noted. This was a qualitative study whose goal was to identify and address the programmatic and policy challenges raised by the integration of NGGS technology into NBS programs. It was not designed to examine the scope and severity of these challenges, but to identify consistent emerging issues for additional investigations. Our participants were leaders and officials within state NBS programs. While beyond the scope of this study, we acknowledge that gathering additional stakeholder viewpoints will also prove critical as NBS programs grapple with the integration of NGGS technology. We chose to focus on the state NBS programs because theirs is often an underrepresented voice in discussions about NBS technology. We believe their perspective is critical to the goal of maintaining the current benefits of NBS, while exploring how genomic screening technologies may be used to enhance or expand those benefits in the genomic era. Given their experiences with the introduction of tandem mass spectrometry, it is understandable that many of the NBS stakeholders would approach NGGS with caution. These experiences also provide a chance to apply the lessons of the past as the programs to the opportunities of the future [25].

As NBS programs continue to evolve, attempts to address challenges must keep child welfare and patient-centered outcomes front and center. This includes ensuring adequate communication between the state programs and primary care providers, ensuring that patients and providers have access to appropriate education and counseling regarding genomic findings, and providing coordination of care with the medical home for follow-up and treatment [26]. The actual impact of NGGS technology will depend in large part on the ability of health departments to address these concerns in comprehensive ways that maintain the current benefits of programs and ensure universal access to screening, while exploring how these screening technologies may also be used to enhance or expand the benefits of newborn screening services [27,28]. If the programmatic and policy challenges from the integration of NGGS technology into NBS programs are not addressed, they could ultimately disrupt the functioning and reduce the benefits of these programs for newborns. Addressing the challenges identified through this project will require further systematic research about the what kinds of genomic information parents may want to know about their newborns, how to effectively communicate those findings, and assess any potential harms associated with receiving genomic screening results. It will also be crucial to address any public policy concerns about the use of genomic data by a public health program.

## Figures and Tables

**Table 1:** List of primary focus group guide questions.

Thematic Area	Specific Questions
Current or expected uses	How have new technologies been integrated into your programs in the past? What is the process? Were any policy changes necessary when these changes were made? Do your programs utilize genomic sequencing technologies now? Have any of your programs had any conversations or made any plans to prepare for genomic technologies and their implications? Have you discussed this as a adjunct technologies to current screening modalities or as a new replacement technology for current MS/MS screening?
Potential Benefits and Challenges	What would you see as the benefits of integrating genomic sequencing into your NBS program? What would you see as the major challenges/barriers of integrating genomic sequencing into your NBS program? Do you think there would be any ethical/social implications of utilizing these kinds of screening tests? What would be the programmatic implications of genomics as an adjunct test? As a replacement test? How do you think genomic information should be communicated to parents? How should they be educated about screening? Do you think that the integration of genomic sequencing should impact the mandatory nature of your programs? Should consent be utilized? How might you implement this kind of authorization?
Educational or communications needs	What kinds of training/education do you think your programs would need to integrate genomics into NBS? What kinds of parental or public education do you think your programs would need to integrate genomics into NBS?
Policy needs	Do you believe your current policies would allow for the integration of genomics into your programs? If not, then what types of policies do you think would be necessary to allow for this kind of integration? What kinds of protections do you think would need to be built into your policies regarding how to collect, store, and manage genomic data?

## Supplementary Materials

Supplementary materials can be found at https://www.mdpi.com/article/10.3390/ijns8010011/s1.
