# Supplementary material for: Genomics and Newborn Screening: Perspectives of Public Health Programs
Source: Int J Neonatal Screen. 2022 Jan 28;8(1):11. doi: 10.3390/ijns8010011 (PMC8883997; doi:10.3390/ijns8010011)
Supplement: Supplementary file 1 [file IJNS-08-00011-s001.zip › IJNS-1515490-supplementary.pdf]

Supplemental Table S1: Additional NBS Program representative quotes regarding NGGS in Newborn Screening

Thematic Area	Quotations
Benefits of using NGGS in NBS	
Reducing Burden of False Positives	Rather than having to stick the baby again, you could use it as a secondary as a screening test. …And that's kind of what we started, because I found that having to call families about probably false-positives and having them go get blood drawn, it just creates a lot of burden and then you have all this excess worry, whereas if we're able to do the gene study and find no mutations and have a repeat on some of these disorders, then we can feel pretty comfortable that that's not it.<br><br>I think they're, you know they're complementary, let's just say, depending on the condition…but you can also get additional information, like perhaps heterozygosity for other genes in a pathway, etc. maybe even explain some of the "false-positives" by looking at targeted whole-exome sequencing. So you know they are complementary.<br><br>I do think probably down the road the cost may become a little more effective and it might help us with false-positives, in reducing false-positives, which might even out cost as well.
Provision of Detailed Risk Information	Let's say for Alpha-thalassemia, we try to follow it along and say 'Well is this likely to be one gene deletion, two gene deletions?' It takes us six months to get a CBC where we're looking for microcytosis. A lot of things could sort of be settled more quickly if we had the sequencing to tell us you know what the actual status is, and maybe some more… I think it might be more commonly found out what certain other variants are and that too could you know settle things, because leaving families hanging with 'Well your child has Hemoglobin F, Hemoglobin A, and Hemoglobin unidentified variant' is very unsatisfying to both the Primary Care Providers and to the families because 'It just seems like there's something not right, but you can't tell us it's definitely okay.' And so I think from the Hemoglobin point of view, some of those things could be settled much more quickly.

Assist with Future Reproductive Decisions	The other thing always in my mind is that when things drag out and you can't settle them, you know mom may get pregnant again and you know it would be so much better, not that I necessarily believe it will change things, but I think it's better from the point of view of counseling to be able to tell them ahead of time 'This is your risk. This is what could happen,' if it's known, but you know when we don't even have a diagnosis or identification of something until the you know baby is six months or a year or older, then you know a lot of them are already pregnant again and might have said 'Well I might…' Not that I know that they would do something different, but just I think giving people the option.
Screen for Disorders Not Otherwise Possible	You know it's just hard to see in that crystal ball there because in a better world, perhaps, where the management of the information is more assured and where things which are now sequences of unknown significance have been the significance has been determined, then it would maybe people would look back and say 'Well that seems reasonable to have everyone sequenced at birth. That way that's the beginning of your permanent medical record, and positive things can be taken from that throughout your entire life, but we're not at that point yet by any means, so and I don't have any real good suggestions of how to approach that or to see what the intermediate steps might be, other than the idea of focusing on what we're mandated to do and trying to do a more thorough and a quicker job from that using molecular information to help.
Equitable Access to Genomic Services	I do want to say that there are potentially families that would like to have access to this type of screening, but I would be uncomfortable having it be part of the mandatory panel. You know we recently have been contacted by several individuals who would like to advocate for us to test for disorders that have not been recommended by the National Secretary's Discretionary Committee, and it's really hard when families have children affected by conditions that they perceive their life could've been better, if it had just been included in that first blood test.
Challenges of Using NGGS in NBS	

Impact of Workforce and Budgets	That can, like we discussed what we would decide is the reportable conditions, because there are actionable conditions, and even if we only took what was on our newborn screening panel and what ACMG has their list, we just don't have the manpower to provide information to Providers and counsel the families, and if Research changes over time, whose responsibility would it be to recontact those families to tell them, 'Oh by the way, this information has changed and we're recontacting you.' We just don't have the workforce that would do that.<br><br>Well I think most states already struggle with long-term follow-up, that there isn't necessarily funding for that, personnel for that, you know, and if we're talking about taking on something so big, I think we still have to even prove that what we do in the long-term already has benefited. Now we can do that in some small ways, but I mean if we're really going to try to convince people to take on something even so much bigger, I think that more funding and more support for long-term follow-up needs to be already in place, and then you know if you can justify more about what we're doing, I mean we all think we're doing something really great and we have bits of proof for it and we have some things we can document in terms of financial savings, but if you're going to try to convince people to invest in this technology in terms of the staffing and the time and the money it will require, I think we have to even step back and say we really need to prove what we already do is so phenomenally great that pursuing something further along the lines might be worth it.<br><br>What we do with diagnostic whole exome, it takes us two to three hours. We write a Consent and follow actually up the next day with another two hours, and sometimes you know the parents give us this blank stare: 'What did you talk about yesterday?' and it's like and you start all over again. You know if this goes to newborn screening, I'm really concerned. Where is the manpower to actually you know do that?<br><br>You know already when we start talking about let's say CF and we screen when the following the algorithm can get up to a panel of 39 mutations, and you know then diagnostically the CF Center may you know send for sequencing of the gene and then comes up with novel variants and nobody knows what to make of it, and the parents get really angry because 'Well you have a result. What does it mean?' And you know when you have to say 'I

	don't know. We don't know,' and you know the idea that you could be opening Pandora's Box to the number of things that we don't know you know what the meaning of it is, but there it is on a piece of paper, I think that amount of staff to just try to field all those questions and come up with a response that satisfied parents could be really (x2) large in number.
Low Genetic Literacy Among Public and Providers:	We even run into situations where the findings are very straightforward and they're very clear and well-understood findings, a child with clear, classical PKU and having a parent that still won't believe it, and so when you just are fighting at that level of you know not only the educational or lack of educational or lack of science understanding or physiology understanding or you know you still have the emotional aspect of things too that interferes with people's ability to understand and deal with it, and I just think you know, boy, in this country, we need a much heavier emphasis on science, life sciences.<br><br>...to think that you're going to go in and try to educate everybody about sequencing, I don't know. It's just kind of a daunting prospect.<br><br>And there's only so much education you can do in Public Health, and we find ourselves repeating the same messages over and over and over again, and after 50 years are still repeating the same messages. So I can't imagine what it's going to take with this whole realm and the diversity of educational backgrounds and understanding in the population is going to be astronomical, I think, to overcome.<br><br>I think that we'd have to really increase, beyond our staff and all of that, we'd really have to increase our efforts of physician education, because results would be coming back and if they're not prepared (and likely they wouldn't be prepared) to deal with what these results are and explain it to their patients, you know you'd have to do some extensive education at least even in the form of 'Well this is who you call when…' so that you know to get some explanation or you know what does this mean for their patient. So I think that the educational outreach would have to be a significant thing. It'd have to be invested in, and it would have to be ongoing.

Lessons of Past Technology Integration	I think the biggest, I mean the big questions are 'Who's going to do it?' and 'How much information are we going to give…?' You know 'How much information is going to come?' I mean Tandem Mass Spec can tell you hundreds of things besides the 26 disorders, but we don't give that information out, so does that apply if you're looking on the DNA level? That's a big question.<br><br>I think a lot of people would admit that some of those shouldn't have made it on the screening panels now, and that was a mistake. So the farther the mistake, you multiply it by a factor of 100, and then the other part is I think you'll open up way more questions than you would answers when doing the screening, and you wouldn't be able to follow up on how to even go after those questions.<br><br>That just reminds me of the Tandem Mass Spec issue when we had incidental findings and then Maine got sued. Now all of a sudden everybody said 'Well if that's going to happen, I guess we have to give them less information.<br><br>Well one of the other issues, and I talked to <PI> about this before, was this is kind of a scenario of Tandem Mass Spectrometry. If you bring it on, do you open everything to detect whatever you can possibly detect, which is the expectation of families, 'If you have the ability, you should be looking for it,' or are you going to window, which is the single gene point, and that puts us in a really tough situation is 'What can we offer?' and 'What can we handle as a program?'
NGGS as Replacement Technology	I mean I really kind of believe it's coming, but I think it's going to be a while. I don't think we're going to just overnight, you know, that Newborn Screening Programs are going to be able to incorporate this. You know maybe it'll start out we'll, like <Participant> was talking about, you know for specific diseases that we already test for, you use it for your second-tier testing, and so I see it more as maybe being kind of an incremental thing that you start to use it for specific applications and then somehow eventually over time maybe it becomes more of a primary kind of screen, but there's a lot of issues around it that have to be worked out before I think you could really start offering it.

	Short-term, I don't see it at all as replacement technology. I think it would be very hard to use as a replacement technology. I see the greatest benefit as possibly bringing on other technologies, and so other conditions… but I see that as also longer-term. Short-term, it appears that you know second tier for sequencing is a reasonable place for it. When you start talking about first-tier sequencing, I just don't see that short-term as a replacement technology. Longer-term I can see that, but I don't really know what that longer term would be. I mean there's a lot of information to still be gathered before we get there, and the ideal is the functional assay, and I still think that's going to be hard to beat.
Original intention of NBS	So one model that I've sort of thought about is if you did exome sequencing, not whole-genome, but the exomes, but only had available data for certain genes, it's probably less involved to do the whole-exome than it is to target a bunch of discreet genes, and if that data were collected early, then it might be available when you have the biochemical results in order to provide second-tier data before reporting. thought when you do a second-tier test, it delays the turnaround time of the primary screen, and that's true, but it may also expedite the time from birth to the time of intervention because there's fewer delays in finding other Specialists. Now it's usually and doctors' appointments and doing the test at a diagnostic lab. So I think whatever, as this whole concept progresses, there's going to be a blurring of the screening and the diagnostic role of the Newborn Screening Lab.<br><br>I just keep thinking newborn screening is meant to catch that baby before they die at like two weeks of age. It's not meant to catch all the other things you're going to find with genome sequencing. That's not the purpose.<br><br>I think that if we used it as a primary mode, we would no longer be Newborn Screening. So I don't think that you would really technically be able to consider it a screening, if you're doing whole-genome sequencing. I don't think we would be doing newborn screening any longer.
